# Thompson’s Compound Self Adjusting Blow Pipe

**Published:** 1853-10

**Authors:** 


### THOMPSON'S COMPOUND SELF ADJUSTING BLOW PIPE.

Dr. J. M. Brown is receiving a lot of these admirable and convenient Blow Pipes, which are for sale on reasonable terms.

